# New strategies for targeting kinase networks in cancer

**DOI:** 10.1016/j.jbc.2021.101128

**Published:** 2021-08-27

**Authors:** Ali E. Yesilkanal, Gary L. Johnson, Alexandre F. Ramos, Marsha Rich Rosner

**Affiliations:** 1 Ben May Department for Cancer Research, University of Chicago, Chicago, Illinois, USA; 2 Department of Pharmacology, University of North Carolina at Chapel Hill, Chapel Hill, North Carolina, USA; 3 Instituto do Câncer do Estado de São Paulo, Faculdade de Medicina and Escola de Artes, Ciências e Humanidades, University of São Paulo, Brazil

**Keywords:** cancer therapy, receptor tyrosine kinases, mitogen-activated protein kinase (MAPK), drug resistance, cell signaling, combination therapy, targeted therapy, inhibitor, mathematical modeling, kinase network, ABL1, Abelson tyrosine-protein kinase 1, ALK, anaplastic lymphoma kinase, BCL2, B-cell lymphoma 2, BCR, breakpoint cluster region, BRD4, bromodomain-containing protein, 4CML, chronic myelogenous leukemia, DDR1, discoidin domain receptor tyrosine kinase 1, EGFR, epidermal growth factor receptor, GIST, gastrointestinal stromal tumor, HER2, human epidermal growth factor receptor 2, IGF1R, insulin-like growth factor 1 receptor, JAK/STAT, janus kinase/signal transducer and activator of transcription, MIB-MS, multiplexed inhibitor beads coupled with mass spectrometry, MEK/ERK, mitogen-activated kinase kinase/extracellular signal regulated kinase, mTOR, mammalian target of rapamycin, PD1, programmed cell death protein 1, PD-L1, programmed death-ligand 1, PI3K/AKT, phosphoinositide 3-kinase/protein kinase B, RKIP, Raf kinase inhibitory protein, TNBC, triple-negative breast cancer

## Abstract

Targeted strategies against specific driver molecules of cancer have brought about many advances in cancer treatment since the early success of the first small-molecule inhibitor Gleevec. Today, there are a multitude of targeted therapies approved by the Food and Drug Administration for the treatment of cancer. However, the initial efficacy of virtually every targeted treatment is often reversed by tumor resistance to the inhibitor through acquisition of new mutations in the target molecule, or reprogramming of the epigenome, transcriptome, or kinome of the tumor cells. At the core of this clinical problem lies the assumption that targeted treatments will only be efficacious if the inhibitors are used at their maximum tolerated doses. Such aggressive regimens create strong selective pressure on the evolutionary progression of the tumor, resulting in resistant cells. High-dose single agent treatments activate alternative mechanisms that bypass the inhibitor, while high-dose combinatorial treatments suffer from increased toxicity resulting in treatment cessation. Although there is an arsenal of targeted agents being tested clinically and preclinically, identifying the most effective combination treatment plan remains a challenge. In this review, we discuss novel targeted strategies with an emphasis on the recent cross-disciplinary studies demonstrating that it is possible to achieve antitumor efficacy without increasing toxicity by adopting low-dose multitarget approaches to treatment of cancer and metastasis.

Cancer is a particularly challenging disease due in large part to heterogeneity and robust compensatory mechanisms. A dynamic disorder that evolves in response to changing environmental conditions, tumors are subject to both genetic and epigenetic alterations that rewire their cellular signaling pathways, giving rise to multiple cellular populations that confer resistance either *de novo* or at a later time in response to treatment. With an exponential increase in our understanding of cellular information transfer in the past few decades, there was an initial optimism that targeted therapy would prove effective as a cure for cancer. Protein kinases, in particular, were among the early favored targets for cancer treatment. However, it is now clear that targeting with single agents is largely ineffective, and even combinations of drugs are problematic for preventing resistance or recurrence.

In particular, use of high-dose drugs either singly or in combination can lead to stimulation of compensatory pathways and networks that promote tumor progression (1, 2, 3, 4). Even combinations of drugs that inhibit single targets across a range of different functional networks can result in toxicity and limit survival (5, 6, 7). Finally, the fact that both normal and tumor cells rely on common signaling pathways is also a limitation to therapeutic efficacy. We need to develop a framework for rationally designing treatments that will work across different tumor cell populations and prevent resistance or recurrence due to activation of alternative signaling networks within cells.

In this review we will discuss recent anticancer strategies and their ability to address preexisting or evolving resistance of tumor cells to treatment. We would suggest that the impediment is not our arsenal of tools to target cancer-promoting genes but instead the way in which we use them. In particular we will focus on a new approach involving the use of low-dose, multidrug combination therapy that targets key kinase signaling networks rather than individual kinases or signaling pathways, as a way of realizing the full potential of combination treatments.

## Single agent approaches

The promise of targeted therapies in cancer was first realized with the discovery of the fusion between *BCR* (breakpoint cluster region gene) and *ABL1* (Abelson tyrosine-protein kinase 1) genes in chronic myelogenous leukemia (CML) patients. Work by Nowell *et al*. (8) and Rowley (9) initially showed that, in many cases, these leukemias are driven by a fusion between *ABL1* and *BCR* genes as a result of a translocation between chromosomes 9 and 22 (also known as the Philadelphia chromosome). The fusion BCR-ABL protein lacks the autoinhibitory domains of the ABL1 kinase and, therefore, results in the constitutive activation of ABL1's proliferative function (10, 11).

Following these observations, the pharmaceutical company Novartis identified the first signal transduction inhibitor Imatinib (also known as STI571 and commercially sold as Gleevec). This drug was shown by Kuriyan and colleagues (12) to bind to the kinase domain of ABL as a competitor of ATP and prevent activation of downstream signaling pathways such as the SRC (SRC proto-oncogene, nonreceptor protein kinase) family, JAK/STAT (janus kinase/signal transducer and activator of transcription), PI3K/AKT (phosphoinositide 3-kinase/protein kinase B), and MEK/ERK (mitogen-activated kinase kinase/extracellular signal regulated kinase) that promote tumorigenesis (11).

Early clinical trials with Imatinib were tremendously successful. The large-scale phase III trial IRIS (The International Randomized Study of Interferon and STI571) comparing single daily dose of Imatinib to IFNα/cytarabine treatment in newly diagnosed chronic myelogenous leukemia patients showed stunning superior efficacy of imatinib over IFNα/cytarabine (18-month complete cytogenetic response rate of 76% in the Imatinib arm *versus* 15% in the IFNα/cytarabine arm) (13). Five-year overall survival rate in this study was 85% and only less than 1% of the patients progressed to accelerated phase or blast crisis phase of the disease 8-year out (14). These results prompted FDA to approve the clinical use of Imatinib in late 2002 as a first-line treatment for newly diagnosed CML patients.

Subsequently, this approval was extended to gastrointestinal stromal tumors (GISTs) that express the receptor tyrosine kinase KIT (tyrosine protein kinase Kit) (15). Today, Gleevec is approved for many other diseases such as myelodisplastic/myeloproliferative diseases in adults, hypereosinophilic syndrome or chromic eosinophilic leukemia, and unresectable and/or metastatic dermatofibrosarcoma protuberans (see the drug label at https://www.accessdata.fda.gov/drugsatfda_docs/label/2020/021588s056s057lbl.pdf).

**Figure 1:**
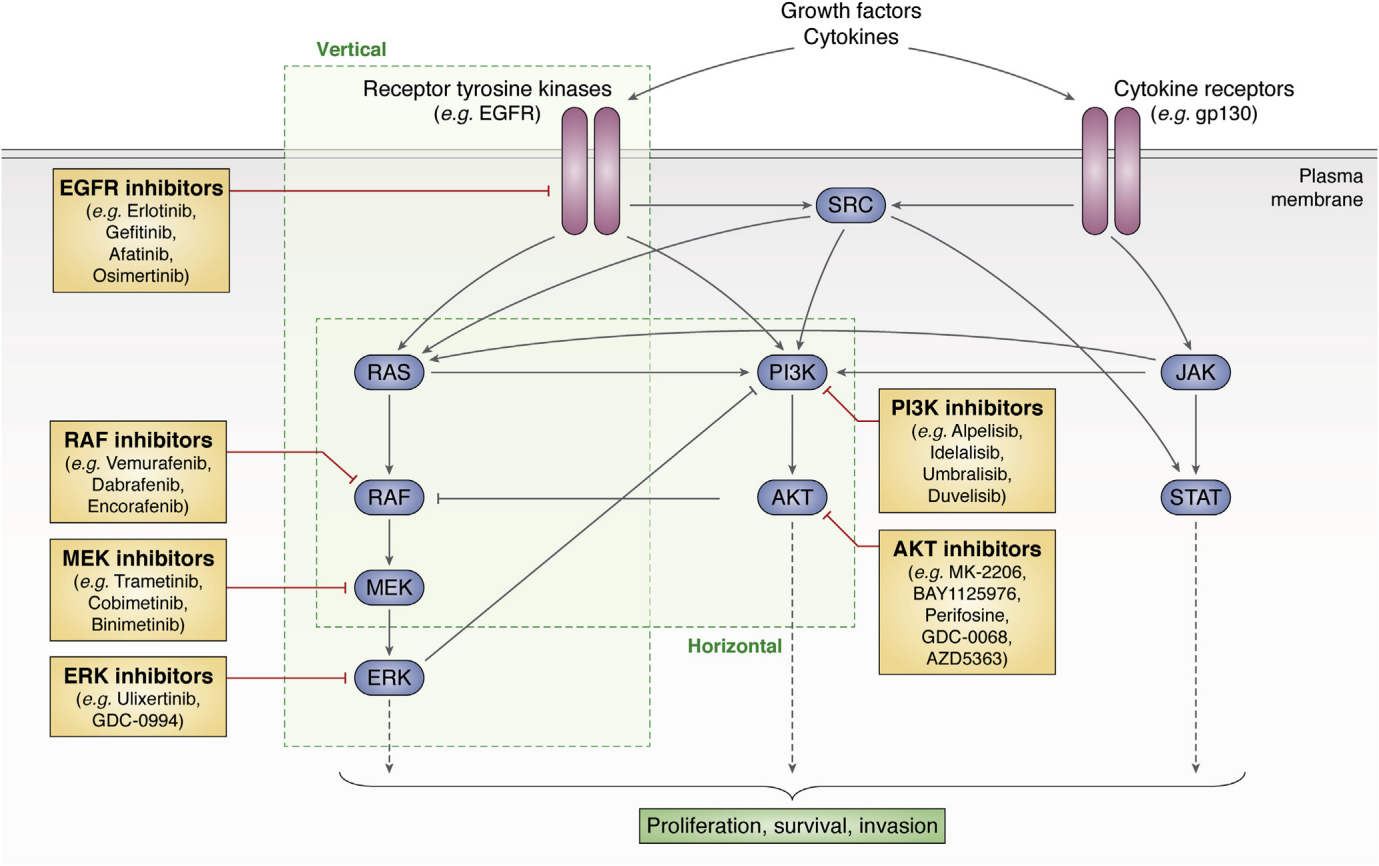
**Extensive cross talk between the oncogenic MAPK, PI3K/AKT, and JAK/STAT signaling pathways and vertical *versus* horizontal modes of inhibition.** Vertical inhibition consists of combining two or more inhibitors targeting the same linear pathway (*e.g.*, an EGFR inhibitor paired with a MEK inhibitor). Positive and negative feedbacks between different components of the MAPK, PI3K/AKT, and JAK/STAT signaling pathways allow for compensatory activation of one pathway when another is inhibited, which leads to tumor resistance. Horizontal inhibition mode aims to solve this resistance problem by targeting different pathways that function in parallel to regulate the same tumor-associated phenotype, such as a MEK inhibitor paired with AKT or PI3K inhibitor.

The success of a single drug targeting a specific oncogenic signaling molecule triggered great excitement in the cancer community. Gleevec was considered to be the “magic bullet” that the cancer community had been searching for since the war against cancer was started in the early 1970s. Following Imatinib's example, a plethora of targeted therapeutic agents, most of which were inhibitors of signaling kinases or receptor tyrosine kinases that drive cancer progression, made it to clinical trials. Some of these trials, particularly the ones testing inhibitors of the oncogenic MAPK pathway (Fig. 1), showed significant improvement with the targeted therapy over the standard of care and, therefore, gained FDA approval.

Sorafenib, an inhibitor of wild-type and V600E-mutant BRAF (V-Raf Murine Sarcoma Viral Oncogene Homolog B), as well as other receptor tyrosine kinases such as VEGFR, has been approved for use in metastatic renal cell carcinoma, hepatocellular carcinoma, and differentiated thyroid carcinoma refractory to radioactive iodine treatment (16, 17, 18). Two other specific inhibitors of BRAF V600E, vemurafenib and dabrafenib, were approved for use in unresectable or metastatic melanoma with the V600E-mutant BRAF (19), reviewed extensively by (20).

EGFR (epidermal growth factor receptor) inhibitors Erlotinib, Gefitinib and Afatinib, as well as Osimertinib, which targets the T970M mutant EGFR, have been approved for use in non-small-cell lung cancers (21). Another EGFR inhibitor, Lapatinib, along with the CDK4/6 (cyclin-dependent kinase 4/6) inhibitors Ribociclib, Palbociclib, and Abemaciclib, has been FDA-approved in breast cancers (https://www.cancer.org/cancer/breast-cancer/treatment/targeted-therapy-for-breast-cancer.html).

ALK (anaplastic lymphoma kinase) inhibitors Crizotinib, Alectinib, Entrectinib, Lorlatinib, Brigatinib, and Ceritinib are all FDA-approved as single agent treatments in non-small-cell lung cancers that are positive for ALK rearrangements (22) (also see https://www.targetedonc.com/view/bazhenova-compares-frontline-alk-inhibitors-for-alk-nsclc). VEGFR (vascular endothelial growth factor receptor) and PDGFR (platelet-derived growth factor receptor) inhibitors Sunitinib, Pazopanib, Cabozantinib, and Axitinib have become widely used in renal cell carcinoma (https://www.cancer.org/cancer/kidney-cancer/treating/targeted-therapy.html).

Tyrosine kinase receptors can also be targeted by monoclonal antibodies that block the activity of the receptor. Anti-HER2 (human epidermal growth factor receptor 2) monoclonal antibody Trastuzumab revolutionized treatment of breast cancers that have ERBB2/HER2 gene amplification (https://www.accessdata.fda.gov/drugsatfda_docs/label/2010/103792s5250lbl.pdf). Anti-EGFR antibody Cetuximab is used in the clinic for the treatment of head and neck cancers and colorectal cancers (https://www.accessdata.fda.gov/drugsatfda_docs/label/2019/125084s273lbl.pdf), while the VEGFR2 antagonist Ramucirumab is used in hepatocellular carcinomas and gastric cancers (https://www.accessdata.fda.gov/drugsatfda_docs/label/2020/125477s034lbl.pdf). These are just a few examples of many targeted small-molecule inhibitors and monoclonal antibody treatments that have demonstrated significant clinical benefit (Fig. 1). A much more comprehensive list of FDA-approved targeted agents can be found in other reviews (22, 23, 24).

Even though a wave of targeted agents has made its way into the clinic after the approval of Gleevec, most targeted treatments have fallen short of the tremendous efficacy and durability of Gleevec. Inhibition of key signaling pathways that drive cancer progression with targeted therapies, while remaining a promising strategy, brought about new fundamental challenges that shifted our understanding of cancer biology. The biggest challenge of single-agent therapies today is the problem of drug resistance and tumor heterogeneity. Most patients who are treated with targeted agents, like the ones mentioned above, either do not respond to the treatment due to tumor intrinsic resistance or develop resistance to the inhibitors over the course of the treatment, eventually succumbing to aggressive disease. Understanding the mechanisms of drug resistance is essential to developing more effective and durable treatment modalities, which has been the focus of extensive research in recent years.

**Figure 2:**
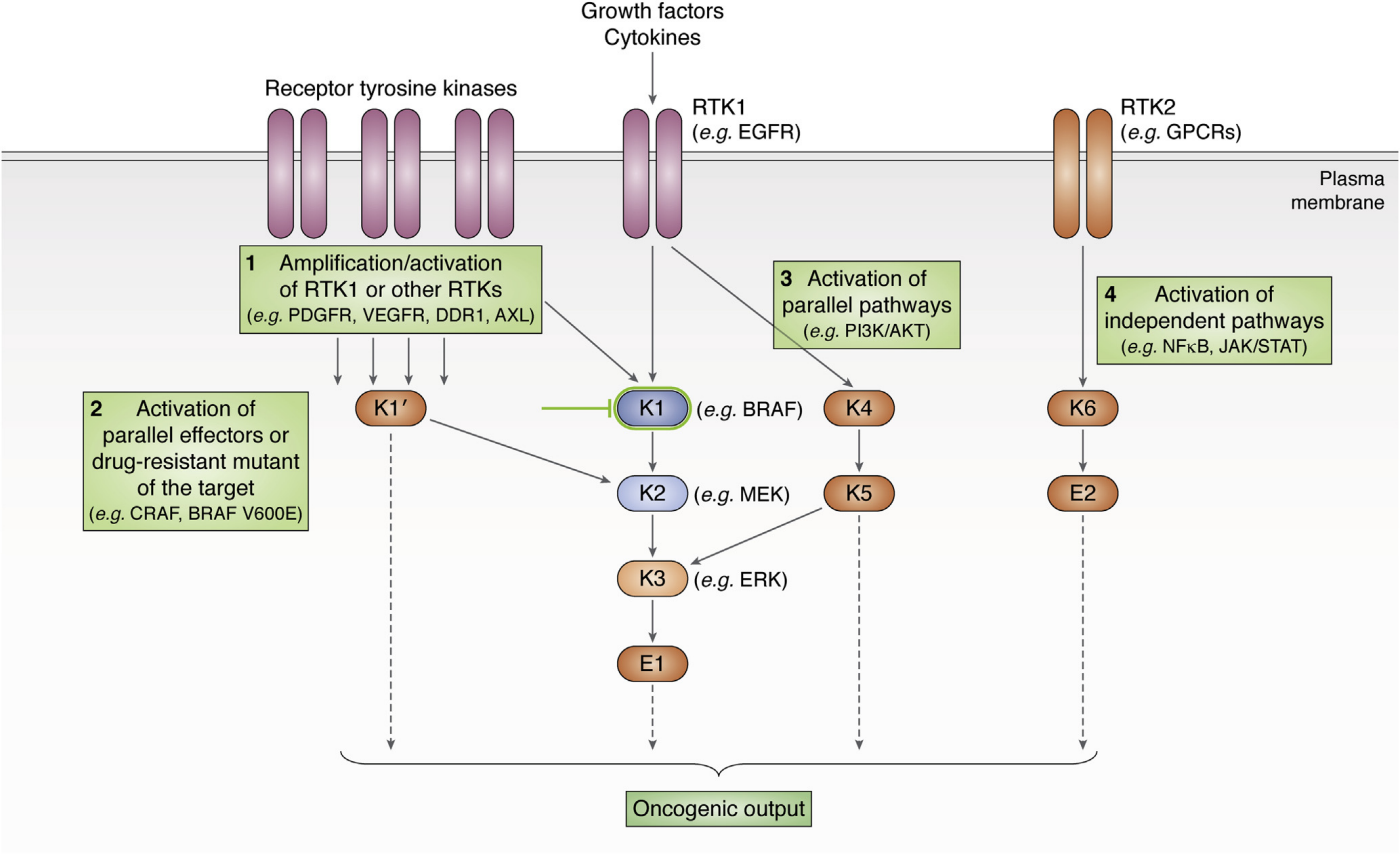
**Mechanisms of drug resistance in cancers.** Oncogenic signaling pathways are composed of receptors (growth factor receptors such as receptor tyrosine kinases, “RTK”s, cytokine receptors, or G-protein-coupled receptors), kinases (“K”), effectors (“E”, *e.g.*, transcription factors) that regulate oncogenic gene expression. Resistance to an inhibitor targeting an oncogenic kinase (indicated by the green blunt-end line targeting K1) can develop by (1) amplification or constitutively activating mutation of upstream receptors that ultimately increase downstream oncogenic signaling and output (indicated by multiple arrows) (2), mutational changes in K1 that makes it resistant to drug binding, or activation of other isoforms of K1 (K1′) that are unaffected by the inhibitor (3), activation of parallel pathways (such as K4/K5 signaling) that can be triggered by the same receptor but bypass the initial pathway (K1/K2/K3) targeted by the drug, or (4) activation of independent pathways that are unaffected by the drug and can achieve similar oncogenic phenotype (RTK2/K6 signaling).

Preclinical and clinical studies have identified multiple mechanisms that lead to resistance to targeted therapies (Fig. 2). Two major mechanisms by which tumors develop resistance are by acquiring secondary mutations in the initial target that renders the inhibitor ineffective or by activating alternative pathways that compensate for the pathway activity that is targeted by the drug. In addition, changes in cell fate, reprogramming of the tumor microenvironment, and immune evasion mechanisms can be adopted by the tumor cells to bypass targeted therapy. Tumors can intrinsically exhibit these resistant states prior to treatment, in which case the patient does not respond to the targeted therapy at all. Alternatively, heterogeneous tumors may harbor drug-resistant subclones that grow out upon selective killing of the sensitive subclones over the course of treatment. In this case, the patient might initially respond to the therapy, but eventually relapses with a more aggressive cancer.

Finally, in recent years multiple studies have demonstrated that targeted agents can actively reprogram the epigenome, transcriptome, or kinome of tumor cells into a more resistant cellular state independently of clonal selection (25). Well-studied cross talk between the MAPK (mitogen-activated protein kinase) and PI3K/AKT pathways has emerged as a prime example of drug resistance by compensatory pathway activation (reviewed in (26)) (Figs. 1 and 2). Both the RAF-MEK-ERK cascade and the PI3K/AKT signaling can be activated by growth factor receptors such as EGFR and regulate processes such as cellular growth and proliferation, survival, and motility (27) (Fig. 1). In many cases, resistance to inhibitors of the RAF-MEK-ERK cascade is mediated by activation of PI3K/AKT signaling (or vice versa) in response to treatment in both clinical and preclinical settings (28) (Fig. 2).

By employing kinomic approaches such as MIB-MS (multiplexed inhibitor beads coupled with mass spectrometry), Duncan *et al*. showed that targeted therapies dynamically reprogram the cancer cell kinome to assume a drug-resistant state (29). Treatment of cell line and genetically modified mouse models of triple-negative breast cancers (TNBCs) with the allosteric MEK inhibitor Selumetinib rewired the tumor cell kinome to activate multiple tyrosine kinases such as PDGFRβ, AXL (AXL receptor tyrosine kinase), VEGFR2, and DDR1 (discoidin domain receptor tyrosine kinase 1) (2). Importantly these changes took place within 1–24 h of treatment, suggesting that the reprogramming was an adaptive response and not the result of selective killing of sensitive subclones of cells. Activation of alternative kinases by Selumetinib was stable and conferred resistance to Selumetinib treatment by sustained prosurvival, progrowth, and angiogenic signaling through the MEK/ERK, PI3K/AKT, and JAK/STAT pathways. In patient-derived xenograft models of TNBC, resistance to the PI3K inhibitor Buparlisib was mediated by activation of NEK9-MAP2K4 (NEMA-related kinase 9—mitogen-activated protein kinase kinase 4) signaling in response to treatment (30).

In a window-of-opportunity trial involving five basal-like TNBC patients and one claudin-low patient, another allosteric MEK inhibitor, Trametinib, induced a strong adaptive bypass response even after 1 week of treatment by upregulating multiple kinases such as FGFR2 (fibroblast growth factor receptor 2), KIT, IGF1R (insulin-like growth factor 1 receptor), and DDR1 (31). The adaptive response was unique to the subtype of tumor and was mediated by genome-wide rewiring of enhancer activity. In particular, Trametinib-treated cancer cells showed *de novo* enhancer formation near the *DDR1* gene that were enriched for BRD4 (bromodomain-containing protein 4) binding, which promoted a drastic increase in DDR1 expression. Trametinib treatment globally doubled the number of active enhancers and nearly tripled the number of superenhancers, while the genes nearby the *de novo* enhancers reflected the specific adaptive response observed in each subtype.

In HER2-positive cell lines of breast cancer, each cell line showed a distinct adaptive response to the HER2 inhibitor Lapatinib by activating a unique set of compensatory kinases through epigenetic means, underscoring the difficulty of identifying effective combinations against widely heterogeneous tumors (32). In the TBCRC 036 window-of-opportunity trial, adaptive response to standard-of-care anti-HER2 treatments (Trastuzumab, Pertuzumab, Trastuzumab+Pertuzumab, or Trastuzumab+Lapatinib) involved activation of compensatory HER3 expression through epigenomic changes in FOXA1 (hepatocyte nuclear factor 3-alpha) binding sites (33).

Similar to these breast cancer examples, in chronic myelogenous leukemias, Imatinib treatment can result in hyperactivation of SRC family kinases such as LYN (v-yes-1 Yamaguchi sarcoma viral related oncogene homolog), as well as other kinases such as MEK, ERK, IKKα (IκB kinase α), PKCβ (protein kinase C beta), and NEK9, ultimately rendering these BCR-ABL-driven cancers unresponsive to Imatinib (34).

**Figure 3:**
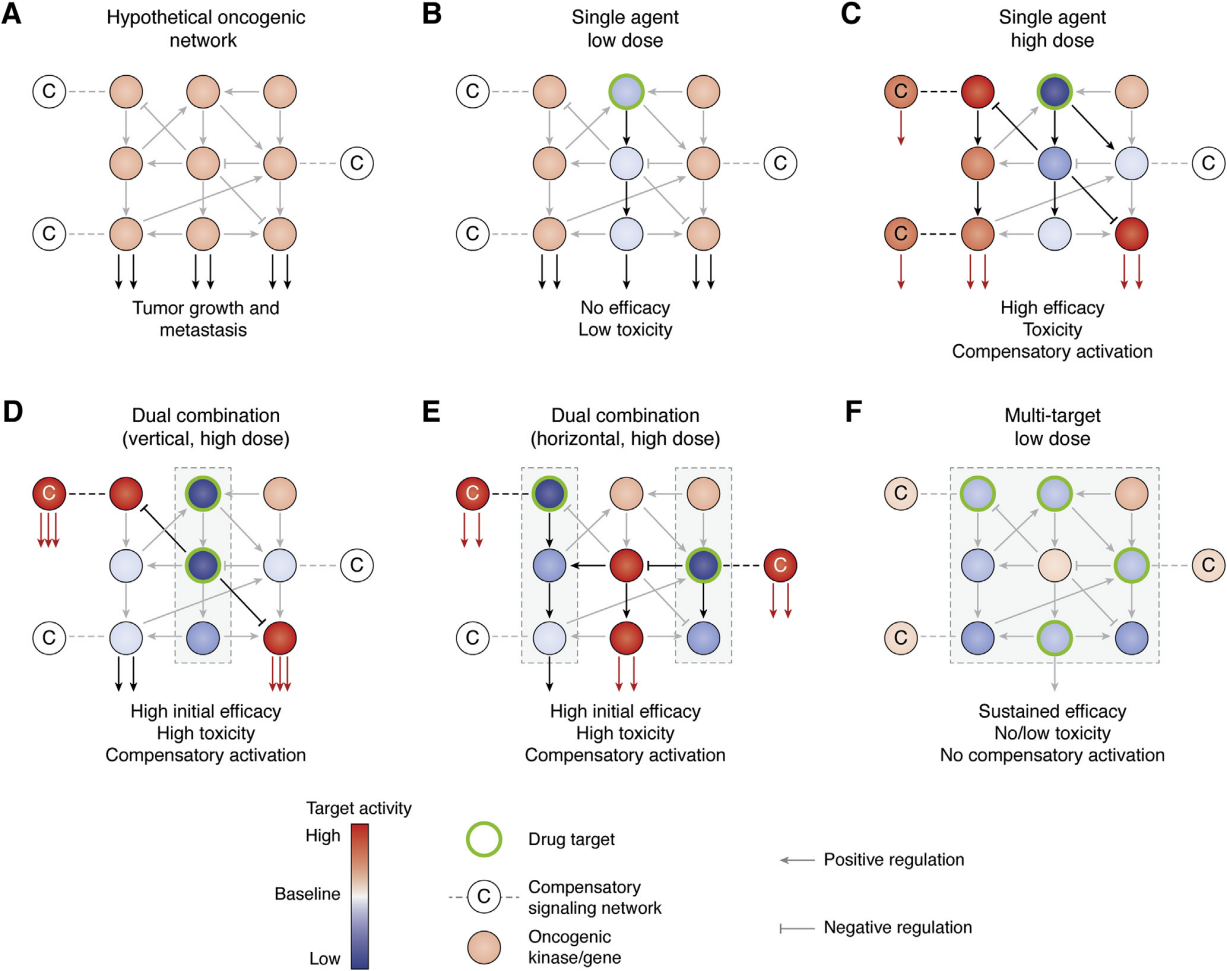
**Strategies for targeting interconnected oncogenic networks using single or multiple targeted agents.***A*, hypothetical oncogenic network that drives tumor growth and metastasis. Each node in the network represents a kinase/gene that interacts positively or negatively with other downstream and upstream nodes within the network. Oncogenic networks are composed of numerous positive and negative feedback mechanisms that constitute an extensive cross talk between different biological pathways. Each node has the potential to activate an alternative compensatory pathway or network due to inherent redundancy in oncogenic signaling pathways. *B*, targeting a single node at a low dose to avoid drug-associated toxicity results in low or no efficacy as the oncogenic signaling is not sufficiently blocked. *C*, high-dose targeting of an individual node can inhibit pathway activity and show initial antitumor efficacy. However, this strategy usually results in activation of alternative pathways and networks that ultimately cause drug resistance. *D*, vertical inhibition of a linear pathway at multiple nodes not only increases drug-associated toxicity, but also increases pressure and resistance mechanisms. The therapeutic efficacy might be stronger in the dual setting, but the activation of adaptive compensatory networks is also more robust. *E*, targeting different pathways with high-dose combinations is still prone to toxicity and resistance as compensatory signaling mechanisms are still activated. *F*, targeting multiple nodes within an oncogenic network at low doses not only effectively reduces the overall oncogenic output of the network, but also prevents activation of compensatory networks and avoids resistance. This strategy also minimizes drug toxicity that usually associated with multidrug combinations since each inhibitor is used at low doses.

The selection pressure and the dynamic reprogramming of cancer cell phenotypes are tightly linked to the dose of the targeted agent being administered. Conventional practice in cancer treatment today follows a “maximum tolerated dose” approach, which aims to administer the highest dose possible where the side effects of the treatment are not too severe or lethal. This dosing strategy is based on the assumption that high-dose treatments will result in higher efficacy and response rates. However, high-dose regimens also cause much stronger selective pressure on intratumoral subclones, ultimately giving rise to a more aggressive subclone (35) (compare Fig. 3, *A* and *C*). Similarly, our team and others have shown that the dynamic reprogramming into a resistance state is much more robust in response to high-dose treatments as opposed to low-dose treatments (2, 36). That being said, low-dose treatment regimens with single targeted agents suffer from the risk of low efficacy (compare Fig. 3, *A* and *B*). Because of these challenges associated with single agent targeted therapies, recent research focused heavily on identifying effective combinations of drugs that can prevent or overcome these resistance mechanisms.

## Vertical inhibition strategies

With an arsenal of hundreds of inhibitors (FDA-approved, in clinical trials, or in development), choosing the right combinations of drugs administered at the right doses to the right group of patients emerges as the new big challenge in cancer treatment (37). This is why many combinational treatment regimens focus on targeting more than one kinase along a convergent pathway to avoid or overcome resistance to disease. This top-down approach of combining inhibitors of kinases belonging to a single key pathway is called “vertical inhibition” (Fig. 1). While the convergent nature of resistance mechanisms (37) can conceptually provide an Achilles heel for cancers, most combinations eventually fail as resistance develops due to the activation of compensatory pathways or mutations in further downstream effectors of the original pathway.

Cancers driven by overactive MAPK signaling are prime example of vertical inhibition strategies as most resistance mechanisms to initial inhibition of this pathway converge on reactivation of its downstream members such as ERK. In colorectal and non-small-cell lung cancers, resistance to EGFR inhibitors develops due to secondary mutations in EGFR (38, 39) or mutations in downstream effectors such as KRAS (Kirsten Rat Sarcoma Viral Oncogene Homolog) or BRAF (40, 41). In these resistant tumors, downstream MEK and ERK signaling is ultimately restored and, therefore, combining EGFR inhibitors with MEK or ERK inhibitors is a viable strategy. Similarly, melanoma cancers that are no longer responsive to BRAF inhibition due to mutations can be resensitized by adding MEK inhibitors to the treatment regimen (42). In fact, BRAF + MEK inhibitor combinations have been FDA-approved standard of care for late stage, unresectable or metastatic melanoma (43).

Other studies demonstrate that combining mTOR (mammalian target of rapamycin) inhibitor rapamycin with AKT inhibitors has a synergistic effect on breast cancer (44), but these combinations have yet to make their way into the clinic. Even though patients may initially benefit from these vertical combination therapies, cancers can come back with activated alternative pathways such as RAS signaling or the receptor tyrosine kinases PDGFR, MET (hepatocyte growth factor receptor) and ERBB3 (V-Erb-B2 Avian Erythroblastic Leukemia Viral Oncogene Homolog 3), or the AKT signaling pathway (45, 46).

A similar kinome adaptive response has been observed in KRAS G12C-mutant tumor cells using irreversible cysteine reactive KRAS G12 inhibitors (47, 48, 49), which specifically interact with the mutant cysteine on the 12th residue of KRAS to inactivate the kinase activity (50). It was discovered that inhibition of KRAS G12C results in an activation of wild-type KRAS, NRAS, and HRAS due to upregulation of receptor tyrosine kinases whose activation overcomes MAPK pathway suppression (47, 48). SHP099, and inhibitor of the SHP2 (Src homology region 2-containing protein tyrosine phosphatase 2) tyrosine phosphatase, revealed that SHP2 is required for receptor tyrosine kinase receptor activation of RAS proteins and activation of the MAPK pathway upon KRAS G12C targeting (51). Combinatorial treatment of cells with a KRAS G12C inhibitor and SHP099 resulted in a more durable inhibition of MAPK pathway reactivation due to loss of receptor tyrosine kinase receptor activation of RAS proteins (47). This represents a different level of vertical inhibition of adaptive bypass response with combination treatment with an inhibitor of receptor tyrosine kinase activation of RAS rather than combinatorial inhibitors of the MAPK pathway.

Another form of vertical inhibition is the targeting of the same molecule with multiple inhibitors. Preclinical studies suggest that, in certain CML cases, combining allosteric BCR-ABL1 inhibitor Asciminib with a tyrosine kinase inhibitor targeting the ATP site of the fusion kinase can be an effective strategy against resistant (52, 53). In HER2 amplified breast cancers and colorectal cancers, an anti-HER2 monoclonal antibody Trastuzumab can be combined with another monoclonal antibody Pertuzumab (54, 55) or with an EGFR/HER2 dual kinase inhibitor Lapatinib (55, 56). Similarly, adding androgen synthesis inhibitor Abiraterone to androgen deprivation therapy shows clinical benefit over androgen deprivation therapy alone in metastatic prostate cancers (57). Unfortunately, these strategies do not provide sustained long-term efficacy either, as cancers eventually become resistant to the vertical inhibition of the oncogenic pathway (55, 58).

There are multiple issues with vertical combination approaches that contribute to the long-term inefficacy of the treatment. First, vertical combinations are very susceptible to additional mutations in downstream effectors of the pathway. A reactivating mutation in a downstream effector can bypass the inhibitors blocking the upstream effectors of the pathway (Fig. 2). Second, vertical combinations suffer from resistance mechanisms that involve activation of alternative pathways (Fig. 3*D*). Clinical applications of vertical combinations still focus on using maximum tolerated doses of each inhibitor, which only increases the selective pressure on the tumor cells. Such high-dose regimens will inevitably result in a resistant cancer that is driven by an alternative pathway that bypasses the initial targeted pathway (Fig. 3*D*). To address the problem of pathway bypassing, recent research has focused on combinational therapies targeting multiple different pathways.

## Horizontal inhibition strategies

Oncogenic pathways are often redundant, and one pathway can compensate for the lack of activity in another pathway by activating similar downstream effectors or by rewiring the tumor cell state. Activation of these compensatory pathways upon targeted therapy constitutes a major mechanism of resistance and a challenging problem for cancer therapy (Fig. 2). In tumors where the driver pathway has an inherent compensatory pathway, it is sensible to molecularly target both pathways together to avoid resistance. This form of combinational strategy that target members of parallel pathways is called “horizontal” inhibition (Fig. 1).

Horizontal inhibition strategy is being widely tested in the context of compensatory activation mechanisms between the MAPK pathway and the PI3K/AKT/mTOR pathway. Since inhibition of one of these pathways results in activation of the other, clinical and preclinical efforts have focused on developing effective combinations that target both of these pathways with the goal of dismantling resistance mechanisms. Early phase clinical trials tested combinations of MEK inhibitors with PI3K inhibitors, mTOR inhibitors, or AKT inhibitors (Fig. 1). BRAF inhibitors with mTOR inhibitors, or even triple inhibition of BRAF + MEK + AKT are also being tested in clinical trials. For example, preclinical studies demonstrated dual inhibition of MEK with PI3K or with PDGFR using brain-penetrant inhibitors is an effective strategy for targeting brain metastases of TNBCs (59). The combination of the MEK inhibitor Trametinib with BET (Bromodomain and Extra-Terminal motif) inhibitors overcomes the adaptive epigenetic reprogramming of the kinome that usually results in resistance to MEK inhibitors in TNBCs (31). A comprehensive discussion of these early phase trials can be found elsewhere (60).

Another rational horizontal combination strategy is to target multiple hallmarks of cancer at once even though the pathways driving each hallmark do not always have compensatory functions. Since Weinberg and Hanahan described key hallmarks of cancer and suggested that combinational treatments should focus on targeting multiple hallmarks (61), many preclinical and clinical studies demonstrated the promise of this approach. Inhibitors of VEGF/VEGFR in combination with EGFR inhibitors in non-small-cell lung cancers constitute a promising strategy that targets tumor angiogenesis as well as uncontrolled growth properties of tumors (62).

Recent studies also showed increased efficacy of anti-PD1 (programmed cell death protein 1)/PD-L1 (programmed death-ligand 1) immunotherapy when combined with blood vessel normalizing agents such as VEGF/VEGFR and angiotensin inhibitors (63). Immune checkpoint inhibitors that block tumor evasion of host immunity are being studied in combination with inhibitors of angiogenesis, based on the hypothesis that increased tumor perfusion allows for increased tumor infiltration of immune cells such as cytotoxic T-cells (64). In July 2020, FDA approved the immunotherapy drug Atezolizumab for combined use with the MEK inhibitor Cobimetinib and RAF inhibitor Vemurafenib in BRAF-mutant melanomas (65). In breast cancers, immunotherapy is being tested in combination with antiproliferative HER-2 inhibitors (66) or DNA repair inhibitors such as PARP (poly ADP-ribose polymerase 1) inhibitors (67) to increase the mutational burden of tumors and make them better targets for the immune system.

In prostate cancers, promising combinations of DNA-damaging PARP inhibitors include inhibitors of androgen receptor signaling, immune checkpoint inhibitors, inhibitors of cell survival mechanisms (*e.g.*, AKT signaling inhibitors), or antiangiogenic agents (reviewed by Pezaro (68)). Inhibitors of antiapoptotic mechanisms such as BCL2 (B-cell lymphoma 2) inhibitors are being tested in combination with other targeted therapies that modify tumor microenvironment and tumor mitochondrial energy metabolism in hematologic malignancies (detailed in (69)).

Though conceptually enticing, horizontal inhibition strategy has many practical challenges. A major problem with the treatment modalities discussed above is the increased toxicity of these treatments. Especially when administered at their maximum tolerated doses, multiple drugs targeting different key pathways can have overlapping toxicity profiles. These toxicities usually reach such severity that the treatment needs to be ceased. Many clinical trials testing combinations of MAPK inhibitors with PI3K/AKT pathway inhibitors have been terminated because of high toxicity and insufficient clinical benefit (70).

Secondly, signaling pathways that drive resistance are usually essential to normal tissue function as well. Therefore, pre-emptively targeting compensatory signaling pathways when the tumor is not yet dependent on them can have unwanted side effects in normal tissues. Identification of cancer-specific mechanisms and dependencies is crucial to developing low-toxicity combinations. Finally, activation of compensatory signaling pathways is still an issue even when multiple pathways (or hallmarks) are targeted at high dose. Even parallel pathways have an abundance of compensatory mechanisms, which can be activated due to robust selective pressure of high-dose inhibition (28) (Fig. 3*E*). Therefore, novel strategies need to reach beyond vertical and horizontal combination approaches and delve into uncharted treatment modalities.

## Low-dose multidrug strategy for effective combinations

Combining multiple inhibitors is clinically challenging because of the increased toxicity associated with these combinations since each drug is used at its maximum tolerated dosage to achieve increased efficacy. Even though there is usually a direct correlation between dosage and efficacy for nontargeted chemotherapeutic drugs, the same relationship does not hold true for targeted agents (71). In some cases, lower doses can be just as effective as higher doses, and higher doses can manifest unpredicted adverse effects without additional clinical benefit (71, 72). Because of these limitations of current combination strategies, recent studies, including our own work, have focused on low-dose multidrug approaches to developing effective cancer treatments.

The premise of low-dose multidrug strategies is based on the idea that we can combine multiple inhibitors to achieve high efficacy without increasing toxicity or triggering resistance by activation of compensatory mechanisms. In this strategy, targeting multiple critical nodes in a disease-driving pathway or network counteracts potential resistance mechanisms as described above (Fig. 3*F*). However, the key feature of this approach is the use of much lower doses than maximum tolerated (*i.e.*, subtherapeutic doses) of the inhibitors. Low-dose combinations can achieve similar therapeutic efficacy to high-dose combinations of the same drugs without exerting high selective pressure on the tumor cells, reducing the risk of developing drug resistance. In addition, toxicity caused by the combination treatment is significantly lower as each inhibitor is used at low doses.

**Figure 4:**
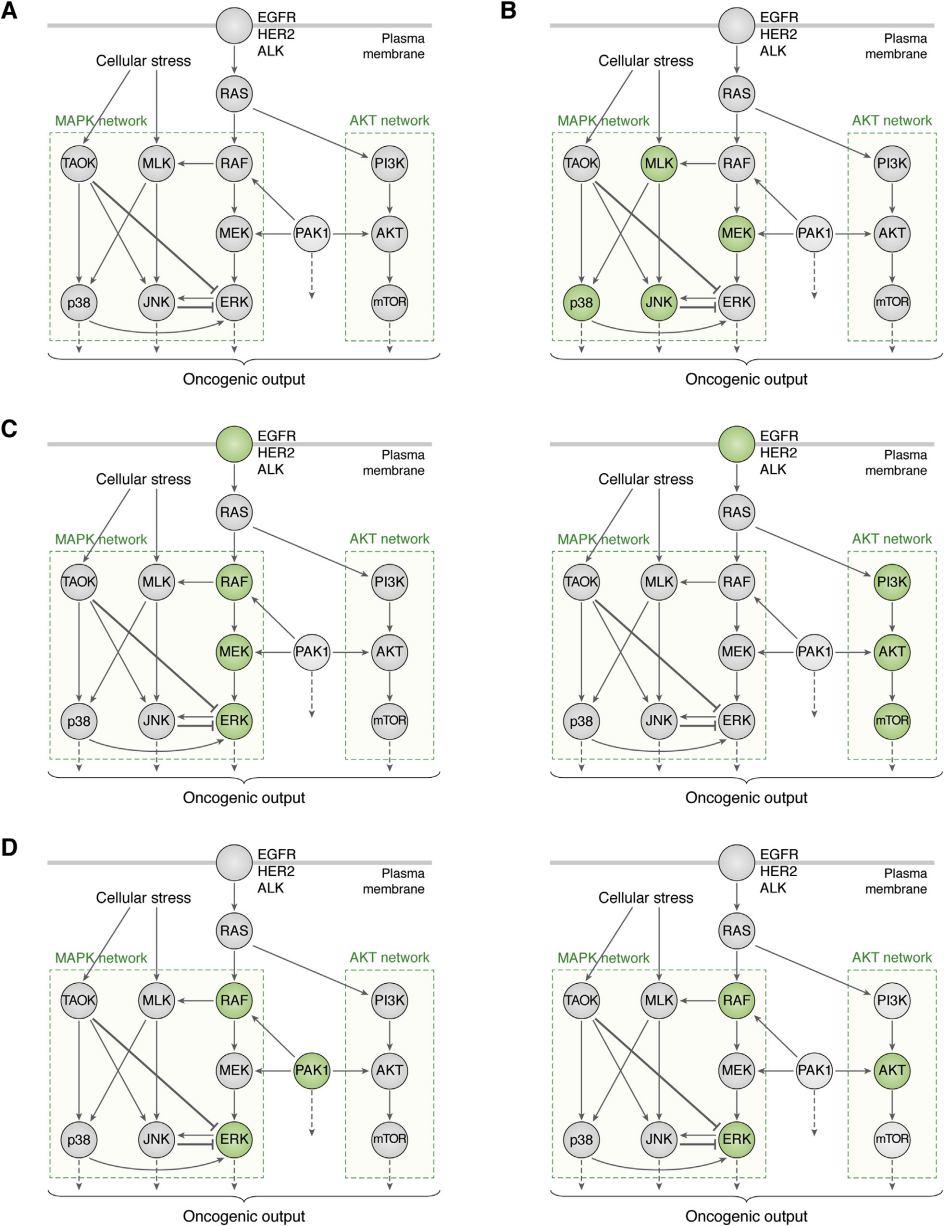
**Summary of effective low-dose multidrug combinations.***A*, simplified network diagram showing the oncogenic MAPK and AKT signaling networks. The nodes displayed indicate the core signaling modules and are not meant to represent the entire network. Each node represents a kinase and the functional relationships between each kinase pair are indicated with arrows (for activation) and blunt-end lines (for inhibition). RAF-MEK-ERK cascade as well as the PI3K-AKT-mTOR cascade can be activated by receptor tyrosine kinases such EGFR, HER2, and ALK or by other kinases such as PAK1. Other components of the MAPK network (*e.g.*, p38 and JNK signaling) can be induced by cellular stress signals such as hypoxia, nutrient deficiency, or unfolded protein accumulation in the endoplasmic reticulum. Panels *B–D* represent recently suggested low-dose multidrug treatment approaches that reduce the oncogenic output of the MAPK and AKT networks. *B*, low-dose combinations suggested by Yesilkanal *et al*.: p38i+JNKi, MEKi+MLKi, or p38i+JNKi+MEKi+MLKi (4D-MAPKi). *C*, low-dose combinations suggested by Neto *et al*.: RAFi+MEKi+ERKi, EGFRi+RAFi+MEKi+ERKi, ALKi+RAFi+MEKi+ERKi, or HER2i+PI3Ki+AKTi+mTORi. D, low-dose combinations suggested by Ozkan-Dagliyan *et al*.: RAFi+ERKi, RAFi+ERKi+PAKi, or RAFi+ERKi+AKTi. TAOK, Thousand and One Amino Acid Protein Kinase.

Recent studies have demonstrated the effectiveness of the low-dose multidrug strategy using the MAPK signaling pathway as an example. Studies by Neto *et al*. and Ozkan-Dagliyan *et al*. identified the low-dose vertical combinations of RAF + MEK + ERK inhibitors, EGFR + RAF + MEK + ERK inhibitors, as well as pan-RAF+ERK inhibitors to be just as effective as high-dose combinations of these drugs in non-small-cell lung cancers (73) and pancreatic cancers (74) (compare Fig. 4*A* with Fig. 4, *C* and *D*). Both research groups performed high-throughput drug combination screens using cell viability and cytotoxicity as their phenotypic target, as well as downstream signaling output of the RAF-MEK-ERK cascade (*e.g.*, pRSK, MYC activation) to monitor the efficacy of tested combinations. Neto *et al*. used IC_20_ values for each inhibitor, the dose at which tumor cell viability was reduced by only 20%. Ozkan-Dagliyan *et al*., on the other hand, tested a wide range of doses of each drug in combination and showed that the synergy between tested drugs was much stronger (measured by Bliss score—a metric for the strength of synergy between two drugs (75)) at low doses than high doses, while the efficacy was comparable between the two dose groups. In fact, Ozkan-Dagliyan *et al*. demonstrated that adding a small dose of ERK inhibitor (80 nM) to pan-RAF inhibitor reduced the dosage of pan-RAF inhibitor required to achieve the same efficacy by 16-fold, suggesting that using high doses is not essential to successful treatment in combinatorial settings.

This finding is in agreement with the clinical observation that adding PI3K inhibitor Copanlisib to the MEK inhibitor Refametinib reduced the maximum tolerated dose and the recommended Phase II dose for each drug in a dose escalation phase I trial in advanced solid tumors (76). The study by Ozkan-Dagliyan *et al*. also demonstrated that, in order to develop an effective vertical combination against MAPK signaling, each inhibitor in the combination must target a different node of the pathway. This is in contrast to the combination strategies that use different inhibitors to target the same molecule, such as the simultaneous use of ATP-site inhibitors and allosteric inhibitors of BCR-ABL1 fusion kinase in CML (53, 77).

A recent study by Yesilkanal *et al*. (36) from our laboratories followed a different approach in identifying effective drug combinations to block metastasis in TNBCs. In this study our goal was to develop a combination treatment against the metastatic phenotype. Therefore, instead of focusing on cytotoxicity or cell viability, we focused on invasion as our primary phenotype. This assay is conceptually critical to developing antimetastatic treatments since the genetics, epigenetics, and microenvironments of the tumor cells differ between metastatic and nonmetastatic states. Our approach involved using kinome analysis to model and pharmacologically phenocopy the actions of a metastasis suppressor called Raf Kinase Inhibitory Protein (RKIP, also known as PEBP1). We identified the stress-induced MAPK network including upstream and downstream regulators of p38, JNK (c-Jun N-terminal kinase), and ERK kinases as a key driver of the metastatic phenotype. By implementing a high-throughput invasion assay as a combinatorial drug screen, we found the dual combinations p38i+JNKi and MEKi+MLKi (MLK: mixed lineage kinase; “i” stands for “inhibitor”), as well as a four-drug combination p38i+JNKi+MEKi+MLKi (termed 4D-MAPKi) to have anti-invasive efficacy without affecting proliferation or cellular viability (compare Fig. 4*A* with Fig. 4*B*), much like the function of the metastasis suppressor RKIP. Notably, the doses of each inhibitor used in these combinations were 4–100-fold lower than their therapeutic doses reported in the literature.

An important finding unique to our study is that low-dose multidrug combinations such as 4D-MAPKi generated a greater homogenous response across multiple cell lines with heterogeneous kinomic and transcriptomic profiles and suppressed activation of alternative signaling networks (36). By using p38, JNK, and ERK activity as the readout of signaling flow through the metastatic stress MAPK network, and the metastatic transcription factor BACH1 (BTB And CNC Homology 1, Basic Leucine Zipper Transcription Factor 1) as the output of the network, we demonstrated that each TNBC cell line tested in our study responded differently to single agent MAPK inhibitors or the dual combinations. This was in part due to striking differences in the topology of the cross talk within the MAPK network in each cell line. 4D-MAPKi treatment, however, effectively reduced both signal flow and the metastatic output of the MAPK network across all cell lines tested. Mathematical modeling of the stress-induced MAPK network with different topological configurations confirmed that 4D-MAPKi was more robust to different topologies, whereas the response to individual inhibitors varied more readily depending on the topology. This observation suggests that low-dose multidrug combinations might provide a solution to clinical heterogeneity in response to targeted treatments across cancer patients.

Importantly, all three studies demonstrated that it is possible to combine multiple targeted agents and achieve high therapeutic efficacy *in vivo* without causing toxicity if the targeted agents are used at low doses instead of their maximum tolerated dose. Ozkan-Dagliyan tested their vertical low-dose RAFi+ERKi combination on a HPAF-II pancreatic xenograft rat model and showed that the combination therapy induced effective regression of established pancreatic tumors and p-RSK (phosphorylated ribosomal S6 kinase) ablation while the rats maintained their overall weight throughout the study, a sign of lack of overall toxicity. Similarly, Neto *et al*. showed that both their three-drug (RAFi+MEKi+ERKi) and four-drug (EGFRi+ RAFi+MEKi+ERKi) combinations were successful at inhibiting tumor growth in subcutaneously or orthotopically injected PDX (patient-derived xenograft) models of EGFRi-resistant NSCLCs (non-small-cell lung cancers) without any apparent weight loss or damage to the gut epithelium or bone marrow. In our TNBC study, the low-dose 4D-MAPKi treated xenografted and syngeneic tumors showed significant therapeutic efficacy even when the treatment was ceased after 3 weeks or the 4D-MAPKi dose was halved. More importantly, metastatic colonization of the cells in the lungs of the mice was almost entirely abolished without any apparent toxicity to the mice. Therapeutic efficacy of these different treatment modalities translated into overall survival benefit in these *in vivo* models of cancer. All three studies showed no cytotoxicity of the low-dose multidrug combinations on normal epithelial cell lines in their respective cancer-related normal tissue.

The most crucial finding from these three studies is the observation that low-dose multidrug targeting of MAPKs did not induce resistance mechanisms in contrast to high-dose targeting of these kinases. In the Neto *et al*. study, cells treated with high-dose EGFR inhibitors Gefitinib or Osimertinib developed resistance, while the low-dose three-drug or four-drug combinations did not. They also demonstrated that PDX tumors *in vivo* were still sensitive to the low-dose combination even after a drug holiday where the treatment was ceased temporarily. Ozkan-Dagliyan *et al*. observed that the RAFi+ERKi combination did not cause reactivation of p-ERK as a resistance mechanism and sustainably downregulated the activity of the downstream mitotic factors MYC (V-Myc Avian Myelocytomatosis Viral Oncogene Homolog), Aurora Kinase, and PLK1 (polo-like kinase 1), though the same combination was associated with increased PAK and AKT activity. The triple combinations of RAFi+ERKi+PAKi and RAFi+ERKi+AKTi prevented compensatory activation of PAK (p21-activated kinase 1) and AKT signaling and further enhanced therapeutic suppression.

Yesilkanal *et al*. showed that the four-drug MAPK inhibitor combination (4D-MAPKi), which targets the stress MAPK network, did not activate the compensatory AKT signaling while maintaining low ERK activity in both cell lines and in syngeneic tumors treated with 4D-MAPKi. Mathematical modeling of the stress-induced MAPK pathway was used to show that partial inhibition of multiple nodes in a driver network with low-dose inhibitor treatments reduces the risk of activating compensatory signaling networks associated with each node, while still effectively inhibiting the output of the entire network. Essentially, the overflow of signal that is blocked by the combination treatment is dissipated across multiple compensatory networks instead of being shunted toward one major compensatory network (such as PI3K/AKT signaling in the case of RAF/MEK/ERK cascade). This analysis also highlights the importance of targeting networks by joining vertical and horizontal inhibition strategies as opposed to targeting linear pathways, because kinases on a linear pathway are more likely to have similar compensatory mechanisms, which can have a compounding effect when inhibited in a vertical fashion.

Given these findings, how can we identify cancer-specific driver networks and predict which novel combinations of targets will yield the most effective and durable treatment of cancers? Neto *et al*. demonstrated that combinations targeting pathways to which the cancer cells are addicted can be effective (73). In cancers driven by ALK fusions such as EML4(Echinoderm microtubule-associated protein-like 4)-ALK(+) lung cancer, ALKi+RAFi+MEKi+ERKi treatment was very effective in suppressing tumor cell growth. In PI3K-addicted HER2(+) breast cancers, a four-drug combination targeting HER2 along with PI3K, AKT, and mTOR successfully blocked tumor cell proliferation.

Yesilkanal *et al*. demonstrated that cancer-specific driver metastatic networks can be identified by identifying the functions of metastasis suppressor proteins that physiologically block metastatic pathways (36). In fact, we found multiple different metastasis suppressors to have a similar transcriptomic output as RKIP suggesting that 4D-MAPKi treatment might be effective against cancers that lack the expression of these physiological suppressors. More importantly, when we compared transcriptomic profiles of multiple cancer types, we found that low RKIP expression usually correlated with high expression of prometastatic genes across different cancer types, suggesting that the RKIP-mimicking 4D-MAPKi treatment can be efficacious in a wide range of cancers. Other systems level approaches based upon bioinformatics as well as mathematical modeling of gene networks will help future studies shed light on novel combination strategies with low toxicities that have wide applications in cancer treatment.

## Informatics and mathematical modeling approaches for combination identification

The design of effective low-dose multidrug therapies for different cancers represents a new class of challenges for theoreticians interested in investigating cancer biology. This is basically a problem of orchestrating the dynamics of a system whose components have a multiplicity of characteristic time scales and chemical affinities. Reorganization of the signaling dynamics through drug treatment is aided by identifying and phenocopying master regulators such as the metastasis suppressor RKIP (78). The existence of a well-worked theoretical toolbox and model system will speed up the field while, eventually, stimulating further insights into modeling and analysis. For example, treatment designers may employ mathematical models and computational simulations to provide further understanding of the dynamics of a functional network and, hence, generate possible strategies to modulate signaling toward a premetastatic or growth-suppressive regimen.

Although phenomenological models may provide invaluable insights into understanding the dynamics of signaling networks, effects such as signal amplification or network stability are dependent on relationships between the kinetic rates of a particular model. Hence, for a particular system under investigation, one needs to use experimental data to establish a sufficiently clear understanding of its properties. Indeed, emergent properties of signaling networks were revealed in a study in which experimentally determined constant rates and concentrations were used to feed a model (79). However, there are conditions in which such a detailed approach is not possible and complementary tools from statistics such as Bayesian inference for estimation of kinetic parameters would be needed (80). Because data from a single experimental design may apply to more than one kinetic model for distinctive signaling pathways, Bayesian inference may be useful as it provides a systematic set of tools for choosing the most appropriate model while reducing the chances of overfitting (81, 82, 83, 84, 85). Developing reliable and efficient computational tools for inference of models for signaling pathways is an active field. Inferring the topology of a signaling network sets an additional complexity layer on this problem, and its solution will require the elaboration of new theoretical methods that provide increased computational efficiency (86).

## Discussion

Therapeutic strategies using targeted agents have evolved since the initial success of Gleevec in CML patients. Based on what we know now about resistance mechanisms to targeted agents and toxicities associated with drug combination, it is clear that the efficacy of Gleevec in BCR-ABL fusion positive tumors was a very special case and not common among cancers. The majority of CML tumors harbor the BCR-ABL fusion kinase and, more importantly, these tumors are relatively homogeneous, allowing for a single agent to achieve full remission in most patients. Subsequent studies demonstrated that other cancers are much more heterogeneous and phenotypically dynamic. Therefore, effective and nontoxic combinations of multiple drugs are needed for successful treatment.

Within the past year, several studies have recognized the efficacy of using low-dose, combinatorial drug treatments for cancer to prevent resistance as well as avoid toxic side effects. Most of the inhibitors target kinases—particularly the MAP kinase network—as key elements of protumorigenic signaling pathways. Furthermore, these studies have demonstrated that this approach is effective against both tumor growth and metastatic progression. In the case of combinatorial drugs targeting metastasis, the treatment would need to be at least a two-step process although in some cases the same drug combination can suppress both cell growth and metastasis. Converting tumor cells to a less metastatic, more epithelial state has the potential to sensitize them to more conventional therapeutic treatments.

An interesting aspect of targeting kinases relates to the potential activation of negative feedback signaling loops that normally modulate kinase activity. To date, clinical treatment usually involves kinase inhibitors that target the ATP binding domain or the catalytic site and are meant to completely suppress catalytic activity. As such, it is likely that these inhibitors induce feedback loops involving direct activation of other compensatory kinase pathways or indirect degradation of transcription factors such as MYC that allow upregulation of receptor tyrosine kinases or other bypass kinases (87). In fact, studies of trametinib, a potent allosteric inhibitor of MEK1/2, at concentrations used in the clinic have led to adaptive responses in the kinomes of patient TNBCs (31). The results we obtained with the multidrug low-dose inhibitor cocktail clearly suppresses signaling but, at least in the case of AKT, is not sufficient to directly activate a bypass kinase pathway. We speculate that this treatment also might avoid transcriptional changes through factors such as MYC that induce compensatory pathways, and this prediction could be addressed through similar experimental studies of other compensatory kinase pathways.

An additional complication would result if we have an integrated system combining negative feedback and external input at the level of upstream kinases. If one were to theoretically model these conditions, the results could differ depending on whether we are considering deterministic or stochastic limits. This point was recently illustrated using a model developed for gene expression (88) that may have sufficient similarities to enable some initial extrapolation to describe kinase signaling systems. Regardless of the approach, questions relating to negative feedback are fundamental to cancer treatment and will need to be addressed in future studies.

However, simply focusing on low-dose drug combinations is not sufficient by itself to avoid triggering recurrence. While at least one recent study aimed to inhibit the linear EGFR-RAS-RAF-MEK-ERK pathway as opposed to single kinases, the study from our group suggests that it is also important to target a key functional network such as the complete MAPK network that includes ERK, JNK, and p38. In this case, cells are not as vulnerable to mutational resistance within a single pathway and are less likely to give rise to compensatory signaling mechanisms. The activity of the underlying functional network (*e.g.*, MAPK) that is required for normal cell survival would be minimally impacted by limiting activity at multiple nodes but should be effective at reducing overall surplus signal. It should be noted that combinations of drugs that each target one node in a different network would also not be as effective as drug combinations that collectively target one network as this would lead to surplus signal in each network and increase the likelihood of compensatory activation. Taken together, these studies provide the promise of a new paradigm in the treatment of tumor growth and metastatic progression in cancer.

Translation of low-dose, multidrug strategies from preclinical studies to the clinic faces a number of future challenges. First, it is important to identify key signaling networks that are required for tumorigenesis and metastatic progression. Second, one needs to identify the combination of drug targets that will enable effective suppression of the network output. Third, the doses for each drug must be as low as possible while still effective in combination and not toxic. Finally, one needs to determine whether different drug combinations are required, how many treatments are sufficient, and in what order. Based on data from both clinical and preclinical studies, targeting the MAPK network would be a reasonable choice for testing this treatment strategy. MAP kinases are required for both tumor growth and stress responses that lead to metastatic progression. Inhibitors of kinases within this network have been extensively used in clinical settings. Thus, one can estimate doses of inhibitors that suppress less than 30% kinase activity through clinical studies such as MEK inhibitor Phase I trials (89). Despite the challenges, both the lack of success using standard of care and accumulating evidence that low-dose, multidrug approaches can be effective with minimal toxic side effects provide a persuasive argument for testing this novel treatment strategy.

## Conflict of interest

This research is also the subject of a pending US patent application #17/048282. The authors declare that they have no conflicts of interest with the contents of this article.
